# Reduced radiation exposure to circulating blood cells in proton therapy compared with X-ray therapy in locally advanced lung cancer: Computational simulation based on circulating blood cells

**DOI:** 10.3389/fonc.2023.1119173

**Published:** 2023-02-27

**Authors:** Nalee Kim, Jungwook Shin, Sung Hwan Ahn, Hongryull Pyo, Jae Myoung Noh, Kyungmi Yang, Woojin Lee, Byoungsuk Park

**Affiliations:** ^1^ Department of Radiation Oncology, Samsung Medical Center, Sungkyunkwan University School of Medicine, Seoul, Republic of Korea; ^2^ Division of Cancer Epidemiology and Genetics, National Cancer Institute, National Institutes of Health, Rockville, MD, United States

**Keywords:** proton beam therapy, lung cancer, lymphopenia, radiation therapy, blood

## Abstract

**Background:**

We estimated the dose of circulating blood cells (CBCs) in patients with locally advanced non-small cell lung cancer for predicting severe radiation-induced lymphopenia (SRIL) and compared pencil-beam scanning proton therapy (PBSPT) and intensity-modulated (photon) radiotherapy (IMRT).

**Materials and methods:**

After reviewing 325 patients who received definitive chemoradiotherapy with PBSPT (n = 37) or IMRT (n = 164). SRIL was diagnosed when two or more events of an absolute lymphocyte count < 200 µL occurred during the treatment course. Dose information for the heart and lungs was utilized for the time-dependent computational dose calculation of CBCs.

**Results:**

The dose distribution of CBCs was significantly lesser in the PBSPT group than that in the IMRT group. Overall, 75 (37.3%) patients experienced SRIL during the treatment course; 72 and 3 patients were treated with IMRT and PBSPT, respectively. SRIL was associated with poor progression-free and overall survival outcomes. Upon incorporating the dose information of CBCs for predicting SRIL, CBC D90% > 2.6 GyE was associated with the development of SRIL with the baseline lymphocyte count and target volume. Furthermore, PBSPT significantly reduced the dose of CBC D90% (odds ratio = 0.11; p = 0.004) compared with IMRT.

**Conclusion:**

The results of this study demonstrate the significance of the dose distribution of CBCs in predicting SRIL. Furthermore, reducing the dose of CBCs after PBSPT minimized the risk of SRIL. Lymphocyte-sparing radiotherapy in PBSPT could improve outcomes, particularly in the setting of maintenance immunotherapy.

## 1 Introduction

Given the physical characteristics of proton and photon (X-ray) beam therapies, proton beam radiation therapy (RT) has intrigued physicians by improving treatment outcomes in patients with non-small cell lung cancer (NSCLC) (1). However, a randomized controlled trial comparing intensity-modulated (photon) RT (IMRT) and proton beam therapy failed to demonstrate clinical benefit in terms of oncologic outcomes and normal tissue toxicities (2). Furthermore, previous retrospective studies showed a trend but no significant benefit in preventing radiation pneumonitis following proton beam therapy (3–5).

Severe radiation-induced lymphopenia (SRIL), which is significant depletion of lymphocytes due to radiation exposure, has been investigated with the emerging interest in immune responses against tumors (6–8). Its clinical significance has been evaluated in various solid tumors (6, 9–12). In this context, we previously showed that pencil-beam scanning proton therapy (PBSPT), an advanced beam delivery technique in proton beam therapy, decreased the occurrence of SRIL (12). Although the etiology of SRIL is multifaceted, the consensus is lacking for dose constraints, which could lead to SRIL because of the lack of tools to compute the dose to circulating lymphocytes.

We previously developed a time-dependent computational framework called the hematological dose (HEDOS), which estimates the dose to circulating blood cells (CBCs) based on a whole-body blood flow simulation and is used to construct the dose–volume histogram of blood cells (bDVH) (13). It has been applied to selective cases. Xing et al. demonstrated the impact of external beam delivery techniques, including IMRT, volumetric-modulated arc therapy, passive proton beam, and PBSPT for the liver treatment plan without clinical data (14). Qian et al. applied HEDOS to correlate the dose to CBCs for patients with metastatic NSCLC, melanoma, or renal cell carcinoma who received immunotherapy and underwent palliative RT (15).

The present study aimed to investigate the clinical effect of the dose information of CBCs on the occurrence of SRIL in patients with NSCLC undergoing concurrent chemoradiotherapy (CCRT) and further determine the association between the occurrence of SRIL and the treatment modality (IMRT or PBPST).

## 2 Materials and methods

### 2.1 Patient population

Upon approval from the institutional review board (approval no.: 2020-01-034), we retrospectively reviewed the data obtained from 325 patients treated with CCRT between November 2016 and December 2019. A total of 124 patients were excluded from the analysis for the following reasons (**Supplementary Figure 1**): lacking information for the dose distribution to the healthy organs (n = 40), missing follow-up data (n = 26), incomplete CCRT course (n = 20), provision of induction chemotherapy before CCRT (n = 18), use of the hybrid IMRT/PBSPT technique (n = 15), and missing weekly blood test reports (n = 5). Finally, the data obtained from 201 patients were analyzed (164 in the IMRT group and 37 in the PBSPT group). The requirement to obtain informed consent was waived because of the retrospective nature of this study.

### 2.2 Treatment

The detailed institutional policies of planning IMRT and PBSPT have been previously described (3). Briefly, based on four-dimensional computed tomography in ten breathing phases, the planning target volume (PTV) was delineated with a 5-mm margin from the clinical target/gross tumor volume. A total dose of 66 GyE in 30 fractions was prescribed in both IMRT and PBSPT groups. **Supplementary Table 1** summarizes the planning criteria for the organs at risk. In the IMRT group, the volumetric-modulated arc therapy was the most frequently used (n = 100, 61.0%), followed by the step-and-shoot method with the 6-MV photon coplanar beam (n = 64, 39.0%). In the PBSPT group, single-field optimization was adopted in 22 (59.5%) patients. The two-field plan was used in 23 (62.2%) patients, and the pencil beam algorithm was applied to all patients. Pinnacle (version 9.2, Royal Phillips Electronics, Miami, FL, USA) and RayStation (RaySearch Laboratories, Stockholm, Sweden) were used for planning IMRT and PBSPT, respectively.

Regarding chemotherapy, 189 (94.0%) patients received six cycles of paclitaxel/cisplatin; six patients received paclitaxel/carboplatin; four patients received gemcitabine/cisplatin; and two patients received combined etoposide and cisplatin. Subsequently, 19 (9.5%) patients received maintenance therapy with durvalumab.

### 2.3 SRIL

Following weekly peripheral blood count assessments during CCRT, lymphopenia was graded based on the Common Terminology Criteria for Adverse Events version 5.00. Based on the absolute lymphocyte count (ALC), SRIL was diagnosed when two or more events of ALC < 200/µL (grade 4) occurred during the CCRT course.

### 2.4 bDVH

We used the pre-generated spatiotemporal blood distribution based on International Commission on Radiological Protection 89th publication that has been described before (13). Dose data of the heart and lungs from each patient’s treatment plan were utilized for HEDOS calculations. The beam-on-time of IMRT and PBPST were both assumed to be 60 s/beam, and the detailed time structure of beam delivery was not considered. We used the dose after all fractions for the analysis. Also, we used 5.3 L for total CBC volume for all patients to compute dose distribution to CBC and thus the volume of one CBC was 0.053 mL (16).

### 2.5 Statistical analysis

Overall survival (OS) rates were calculated from the first date of CCRT to the date of death or last follow-up. Progression-free survival (PFS) rates were calculated from the first date of CCRT to the date of progression, death, or the last follow-up. Baseline characteristics were evaluated using the chi-squared test or Fisher’s exact and Mann–Whitney U tests to assess categorical and continuous variables, respectively. The Kaplan–Meier method and log-rank test were used for OS and PFS. A Cox regression model was used for the multivariable analysis of factors affecting OS and PFS that had a *p*-value < 0.05 in the univariable analysis. The logistic regression analysis was performed to evaluate the predictive factors of SRIL. The factors were selected in stepwise regression after ten-fold cross-validation and included in the multivariate analysis of SRIL. To identify the optimal cutoffs for dosimetric parameters of CBCs, maximally selected rank statistics were performed. A null multivariate model was built based on patient and tumor characteristics. The Akaike information criterion (AIC) was used to compare multivariate models to select the most discriminative dosimetric predictor of SRIL. Statistical significance was set at a two-tailed *p*-value < 0.05. All statistical analyses were performed using SPSS version 25.0 (IBM Corp., Armonk, NY, USA) and R version 4.0.2 (R Foundation for Statistical Computing, Vienna, Austria).

## 3 Results

### 3.1 Patient population

**Figure 1:**
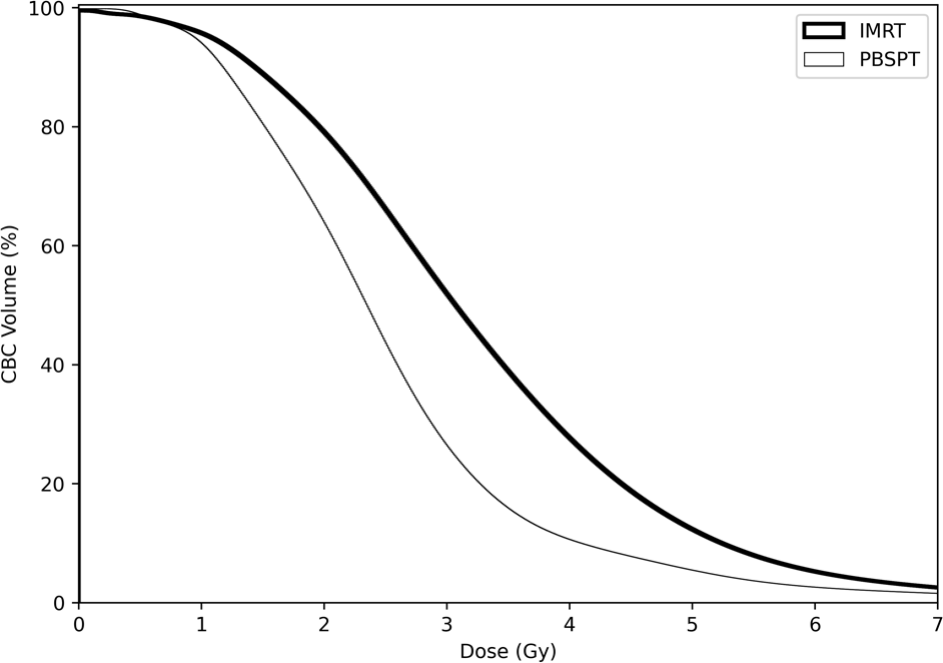
Dose-volume parameters of circulating blood cells in both treatment modalities for all patients.

Overall, compared with the patients in the PBSPT group, patients in the IMRT group were younger, more frequently diagnosed with adenocarcinoma, and had a more advanced nodal disease (**Supplementary Table 2**). Although the target volume or prescription dose did not differ between the two groups (PBSPT and IMRT groups), the dose parameters for V5GyE, V10GyE, and V20GyE of the lungs, mean lung dose, V30GyE of the heart, and mean heart dose were significantly lower in the PBPST group than those in the IMRT group (**Table 1**). Moreover, bDVH was significantly lower in the PBPST group than that in the IMRT group (**Table 1**). **Figure 1** shows bDVHs for the entire population stratified by RT modality.

**Table 1:** Detailed information on dose parameters according to the radiation therapy modality.

		Total	IMRT	PBSPT	*p*-value
		n = 201	n = 164	n = 37	
GTV, cc		108.9 [67.4–203.4]	109.4 [67.9–205.8]	104.7 [66.0–194.1]	0.966
CTV, cc		314.4 [208.1–495.2]	310.2 [202.0–489.2]	350.2 [232.9–520.4]	0.269
PTV, cc		575.8 [387.4–805.9]	572.6 [385.9–792.5]	592.3 [389.3–890.4]	0.522
CTV V100%, %		96.3 [95.0–97.8]	96.2 [95.0–97.2]	99.0 [96.0–99.1]	<.001
PTV V95%, %		97.1 [94.1–98.8]	97.1 [94.2–99.0]	96.8 [94.1–98.4]	0.174
Radiotherapy	Total dose, GyE	66.0 [66.0–66.0]	66.0 [66.0–66.0]	66.0 [66.0–66.0]	0.769
	BED, Gy	80.5 [80.5–80.5]	80.5 [80.5–80.5]	80.5 [80.5–80.5]	0.997
Lungs	V_5GyE_, %	51.2 [42.6–61.3]	54.9 [47.2–63.2]	35.2 [27.2–41.5]	<0.001
	V_10GyE_, %	40.9 [33.8–47.1]	43.4 [36.2–49.7]	30.7 [23.7–36.1]	<0.001
	V_20GyE_, %	31.3 [24.1–36.5]	32.3 [26.4–37.4]	23.5 [20.1–27.5]	<0.001
	Mean dose, GyE	17.6 [14.3–20.6]	19.0 [15.3–21.3]	13.9 [11.1–16.2]	<0.001
Heart	V_30GyE_, %	12.6 [5.9–26.3]	14.6 [5.7–28.2]	9.1 [6.0–12.9]	0.013
	V_45GyE_, %	7.2 [2.9–16.2]	8.0 [2.9–17.4]	5.4 [3.0–8.5]	0.090
	Mean dose, GyE	12.0 [6.0–18.9]	13.3 [7.1–21.5]	7.5 [5.1–10.3]	<0.001
CBC	Mean, GyE	2.93 [2.22–3.93]	3.10 [2.29–4.09]	2.39 [1.76–3.02]	0.002
	D10, GyE	3.49 [2.66–4.64]	3.66 [2.73–4.76]	2.94 [2.12–3.64]	0.003
	D20, GyE	3.30 [2.50–4.36]	3.47 [2.58–4.52]	2.74 [1.99–3.43]	0.003
	D30, GyE	3.16 [2.39–4.19]	3.32 [2.47–4.35]	2.60 [1.90–3.25]	0.002
	D40, GyE	3.03 [2.30–4.05]	3.20 [2.37–4.21]	2.48 [1.82–3.12]	0.002
	D50, GyE	2.92 [2.21–3.92]	3.09 [2.29–4.08]	2.38 [1.76–3.01]	0.002
	D60, GyE	2.81 [2.12–3.78]	2.98 [2.20–3.95]	2.29 [1.68–2.89]	0.002
	D70, GyE	2.70 [2.04–3.63]	2.83 [2.11–3.80]	2.19 [1.61–2.77]	0.001
	D80, GyE	2.57 [1.93–3.46]	2.70 [2.00–3.61]	2.07 [1.53–2.64]	0.001
	D90, GyE	2.39 [1.79–3.22]	2.52 [1.86–3.33]	1.91 [1.41–2.45]	<0.001
CBC	V_0.5GyE_, %	100.0 [100.0–100.0]	100.0 [100.0–100.0]	100.0 [100.0–100.0]	0.290
	V_1.0GyE_, %	100.0 [100.0–100.0]	100.0 [100.0–100.0]	100.0 [99.9–100.0]	<.001
	V_1.5GyE_, %	100.0 [98.53–100.00]	100.00 [99.22–100.00]	99.2 [82.7–100.0]	<.001
	V_2.0GyE_, %	98.9 [73.7–100.0]	99.5 [80.4–100.0]	85.2 [19.3–99.1]	<.001
	V_2.5GyE_, %	84.4 [20.1–99.7]	91.0 [26.8–99.8]	38.2 [0.6–87.7]	0.001
	V_3.0GyE_, %	42.4 [1.3–95.9]	57.7 [2.2–96.9]	7.8 [0.0–50.5]	0.002
	V_3.5GyE_, %	9.7 [0.0–77.7]	18.3 [0.0–84.6]	0.5 [0.0–16.2]	0.002

Values are expressed as median [interquartile range].

IMRT, intensity-modulated radiation (photon) therapy; PBSPT, pencil-beam scanning proton therapy; GTV, gross tumor volume; CTV, clinical target volume; PTV, planning target volume; GyE, gray equivalent; BED10, biological effective dose with α/β of 10; Vxx%, volume receiving more than proportion to prescribed dose; Dxx, dose to XX% of volume; V_XXGyE_, volume receiving over XX GyE; CBC, circulating blood cell.

### 3.2 SRIL

**Figure 2:**
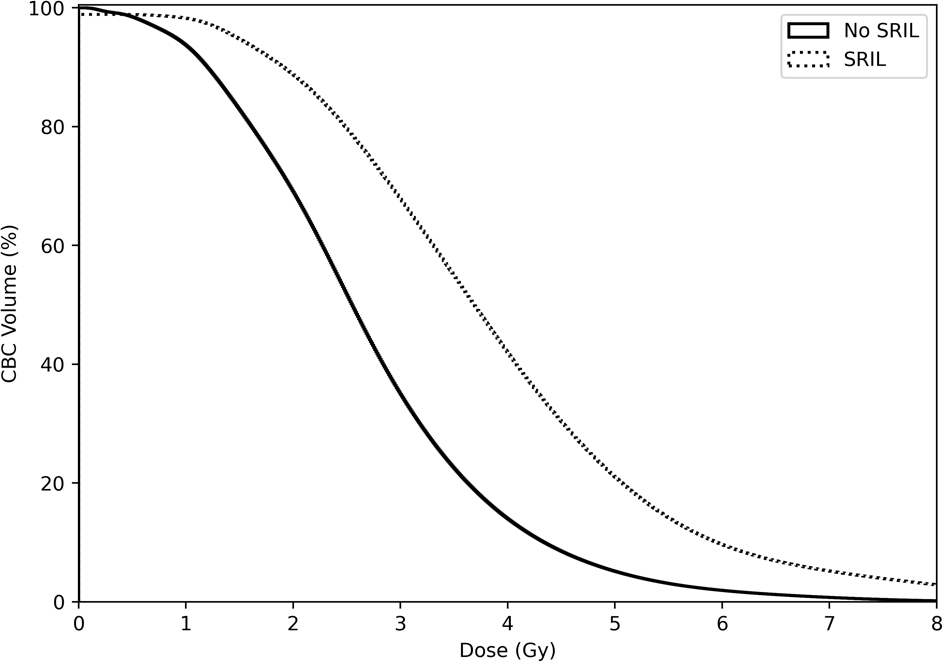
Dose-volume parameters for circulating blood cells stratified by severe radiation-induced lymphopenia.

ALC in the entire cohort decreased gradually during the CCRT course and recovered afterward (**Supplementary Figure 2**). From week 4 to the last week of the CCRT course, ALCs were significantly lower in the IMRT group than in the PBSPT group (**Supplementary Figure 2**, **Supplementary Table 3**). Among 107 (53.2%) patients who developed grade 4 lymphopenia during the CCRT course, 75 (37.3% of entire patients) experienced SRIL, including three (8.1% of PBPST group) in the PBPST group and 72 (43.9% of IMRT group) in the IMRT group (*p* < 0.001). In addition, none of the patient or tumor characteristics, except for baseline ALC values, differed between the two groups. Patients with SRIL showed significantly lower baseline ALC than those without SRIL (median, 2,010/µL vs. 2,140/µL, *p* = 0.029, **Table 2**). Regarding dose-volume parameters, patients with SRIL had a larger target volume and showed a higher dose distribution to the lung and heart than those without SRIL (all *p* < 0.001, **Table 2**). Moreover, bDVH for patients with SRIL also differed from that for patients without SRIL (**Figure 2**).

**Table 2:** Patient, tumor, and treatment characteristics stratified by severe radiation-induced lymphopenia. .

Patient and tumor characteristics		SRIL	No SRIL	
		n = 75	n = 126	*p*-value
Sex	Female	18 (24.0)	24 (19.0)	0.404
	Male	57 (76.0)	102 (81.0)	
Age		63 [57–68]	64 [58–69]	0.340
ECOG	0	6 (8.0)	22 (17.5)	0.061
	1 or 2	69 (92.0)	104 (82.5)	
Smoking history	Never-smoker	18 (24.0)	21 (16.7)	0.204
	Ex- or current-smoker	57 (76.0)	105 (83.3)	
Tumor laterality	Left	25 (33.3)	53 (42.1)	0.110
	Right	49 (65.3)	66 (52.4)	
	Central	1 (1.3)	7 (5.6)	
Tumor location	Upper lobe	42 (56.0)	69 (54.8)	0.226
	Middle lobe	5 (6.7)	18 (14.3)	
	Lower lobe	28 (37.3)	39 (31.0)	
Pathology	Non-ADC	36 (48.0)	57 (45.2)	0.704
	ADC	39 (52.0)	69 (54.8)	
T-stage	cT1 or cT2	43 (57.3)	81 (64.3)	0.327
	cT3 or cT4	32 (42.7)	45 (35.7)	
N-stage	cN2	24 (32.0)	46 (36.5)	0.516
	cN3	51 (68.0)	80 (63.5)	
Clinical stage	IIIA	7 (9.3)	25 (19.8)	0.086
	IIIB	55 (73.3)	75 (59.5)	
	IIIC	13 (17.3)	26 (20.6)	
Baseline ALC,	(×10^3^/μL)	2.01 [1.48–2.33]	2.14 [1.68–2.60]	0.029
Baseline ANC	(×10^3^/μL)	4.82 [3.74–6.40]	5.01 [3.47–5.99]	0.973
Details of radiation therapy				
Modality	IMRT	72 (96.0)	92 (73.0)	<.001
	PBSPT	3 (4.0)	34 (27.0)	
GTV, cc		171.8 [93.4–275.2]	89.7 [46.8–154.0]	<.001
CTV, cc		441.4 [283.4–613.4]	264.9 [174.6–411.7]	<.001
PTV, cc		739.5 [531.2–938.5]	471.4 [339.1–707.6]	<.001
Total dose, GyE		66.0 [66.0–66.0]	66.0 [66.0–66.0]	0.782
BED10, Gy		80.5 [80.5–80.5]	80.5 [80.5–80.5]	0.960
Lung	V_5GyE_, %	58.4 [51.2–64.5]	47.0 [36.4–58.4]	<.001
	V_10GyE_, %	44.7 [40.9–48.8]	37.7 [30.7–45.9]	<.001
	V_20GyE_, %	33.8 [29.9–38.5]	28.2 [22.3–33.4]	<.001
	Mean dose, GyE	20.1 [17.1–22.3]	16.2 [13.1–19.4]	<.001
Heart	V_30GyE_, %	21.4 [10.8–35.0]	9.5 [3.5–19.5]	<.001
	V_45GyE_, %	13.0 [5.6–18.6]	5.3 [1.5–11.2]	<.001
	Mean dose, GyE	9.4 [3.9–15.6]	4.0 [1.2– 7.7]	<.001
CBC	Mean, GyE	3.81 [2.80–4.81]	2.55 [1.86–3.34]	<.001
	D10, GyE	4.53 [3.34–5.72]	3.05 [2.23–3.92]	<.001
	D20, GyE	4.28 [3.15–5.40]	2.87 [2.10–3.72]	<.001
	D30, GyE	4.11 [3.01–5.17]	2.75 [2.01–3.58]	<.001
	D40, GyE	3.96 [2.90–4.98]	2.64 [1.93–3.45]	<.001
	D50, GyE	3.79 [2.79–4.80]	2.54 [1.85–3.32]	<.001
	D60, GyE	3.66 [2.69–4.62]	2.45 [1.78–3.14]	<.001
	D70, GyE	3.52 [2.59–4.43]	2.35 [1.69–3.02]	<.001
	D80, GyE	3.36 [2.46–4.21]	2.21 [1.60–2.87]	<.001
	D90, GyE	3.14 [2.30–3.89]	2.05 [1.48–2.67]	<.001

Values are expressed as number of patients (%) or median [interquartile range].

ECOG, Eastern Cooperative Oncology Group; ADC, adenocarcinoma; ALC, absolute lymphocyte count; ANC, absolute neutrophil count; IMRT, intensity-modulated radiation (photon) therapy; PBSPT, pencil-beam scanning proton therapy; GTV, gross tumor volume; CTV, clinical target volume; PTV, planning target volume; GyE, gray equivalent; BED10, biological effective dose with α/β of 10; Dxx, dose to XX% of volume; VXXGyE, volume receiving over XX GyE; CBC, circulating blood cell.

### 3.3 Prognostic value of SRIL

**Figure 3:**
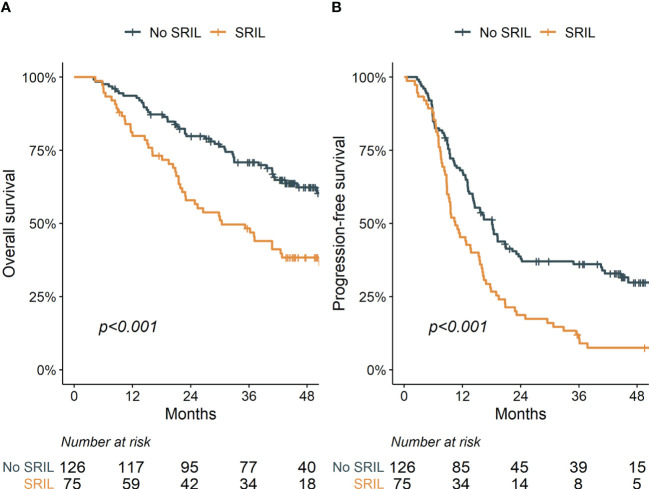
Overall **(A)** and progression-free **(B)** survival stratified by severe radiation-induced lymphopenia.

With a median follow-up of 39.8 (IQR [interquartile range], 21.0–49.4) months, the 3-year OS and PFS rates were 62.4% and 26.2% for the entire cohort, respectively. Patients with SRIL showed poorer OS and PFS outcomes than those without SRIL (3-year OS, 48.3% vs. 70.9%, *p* < 0.001; 3-year PFS, 10.5% vs. 36.1%, *p* < 0.001, **Figures 3A, B**). The multivariable analysis revealed that SRIL remained a significantly unfavorable factor for both OS and PFS (**Table 3**).

**Table 3:** Prognostic factors for overall and progression-free survival.

Overall survival		Univariable analysis			Multivariable analysis		
Variables		HR	95% CI	*p*-value	HR	95% CI	*p*-value
Treatment modality	(IMRT vs. PBSPT)	0.84	0.46–1.50	0.551			
Sex	(Female vs. male)	1.62	0.94–2.78	0.083			
Age	(<65 vs. ≥ 65 years)	1.18	0.78–1.77	0.442			
Histology	(non-ADC vs. ADC)	0.52	0.35–0.79	0.002	0.61	0.40–0.93	0.021
Clinical T-stage	(T1–2 vs. T3–4)	1.18	0.78–1.79	0.444			
Clinical N-stage	(N2 vs. N3)	0.73	0.48–1.12	0.151			
GTV	Continuous (per 10cc)	1.33	1.12–1.57	0.001	1.01	1.00–1.02	0.142
Total dose	(>66 vs. ≤66 GyE)	1.56	0.72–3.37	0.263			
BED10	(<80 vs. ≥80 GyE)	1.20	0.73–1.95	0.469			
Baseline ALC	(continuous)	0.41	0.20–1.11	0.137			
SRIL	(No vs. yes)	1.54	1.02–2.33	0.040	1.42	1.01–2.25	0.043
Progression-free survival		Univariable analysis			Multivariable analysis		
Variables		HR	95% CI	*p*-value	HR	95% CI	*p*-value
Treatment modality	(IMRT vs. PBSPT)	0.57	0.36–0.91	0.019	0.75	0.45–1.22	0.244
Sex	(Female vs. male)	0.84	0.57–1.21	0.344			
Age	(<65 vs. ≥ 65 years)	0.63	0.45–0.87	0.005	0.68	0.48–0.94	0.021
Histology	(non-ADC vs. ADC)	1.08	0.78–1.48	0.651			
Clinical T-stage	(T1–2 vs. T3–4)	0.98	0.71–1.35	0.881			
Clinical N-stage	(N2 vs. N3)	1.11	0.79–1.55	0.550			
GTV	Continuous (per 10 cc)	1.01	1.00–1.02	0.142			
Total dose	(>66 vs. ≤66 GyE)	1.09	0.63–1.88	0.768			
BED10	(<80 vs. ≥80 GyE)	1.43	0.98–2.10	0.065			
Baseline ALC	(Continuous)	0.75	0.60–0.93	0.009	1.33	0.91–1.93	0.139
SRIL	(No vs. yes)	1.91	1.39–2.64	<.001	1.65	1.17–2.33	0.004

*The foreparts of parentheses were set as the reference group.

HR, hazard ratio; CI, confidence interval; IMRT, intensity-modulated radiation (photon) therapy; PBSPT, pencil-beam scanning proton therapy; ADC, adenocarcinoma; GTV, gross tumor volume; GyE, gray equivalent; BED10, biological effective dose with α/β of 10; ALC, absolute lymphocyte count; SRIL, severe radiation-induced lymphopenia.

### 3.4 Factors predicting SRIL

First, we performed a multivariate analysis to predict the development of SRIL based on clinical and treatment factors other than dose information of CBCs. Both baseline ALC (odds ratio [OR] = 0.60, 95% confidence interval [CI]: 0.37–0.94, *p* = 0.026) and PTV (OR = 1.02, 95% CI: 1.01–1.03, *p* = 0.001) were related to an increased risk of SRIL (**Table 4**, **Supplementary Table 4**). AIC in the null model, including baseline ALC and PTV, was 245.52 (**Supplementary Table 5**). Subsequent analysis showed that bDVH was related to an increased risk of SRIL in the univariate analysis (**Supplementary Table 4**). In the stepwise regression model, CBC of D90% as the continuous variable (OR = 3.25, 95% CI: 1.98–5.67, *p* < 0.001) was the only significant factor among bDVH along with baseline ALC (OR = 0.46, 95% CI: 0.27–0.76, *p* = 0.004, **Table 4**).

**Table 4:** Multivariate analysis to predict severe radiation-induced lymphopenia.

Variables		Multivariate analysis		
		OR	95% CI	*p*-value
Model 1 (without CBC data)				
Baseline ALC	(Continuous)	0.60	0.37–0.93	0.026
PTV	(Continuous, per 10 cc)	1.02	1.01–1.03	0.001
Model 2 (Stepwise model)				
Baseline ALC	(Continuous)	0.47	0.27–0.78	0.005
PTV	(Continuous, per 10 cc)	1.01	1.00–1.02	0.049
CBC D90, GyE	(Continuous)	2.13	1.54–3.06	<.001
Model 3 (AIC comparison)				
Baseline ALC	(Continuous)	0.48	0.28–0.78	0.004
PTV	(Continuous, per 10 cc)	1.01	1.00–1.02	0.022
CBC D90, GyE	(≤ 2.6 vs. > 2.6 GyE)	6.38	3.19–13.26	<.001

*The foreparts of parentheses were set as the reference group.

CBC, circulating blood cells; ALC, absolute lymphocyte count; PTV, planning target volume; D90, dose to 90% of volume; GyE, gray equivalent; AIC, Akaike information criterion.

Finally, we separately generated models with each significant dosimetric variable of CBCs together with the null model. After comparing AIC, the model including CBC D90% > 2.6 GyE was associated with the lowest AIC at 218.46 (**Supplementary Table 5**) and remained significant in the multivariate analysis (OR = 6.38, 95% CI: 3.19–13.26, *p* < 0.001), along with baseline ALC and PTV (all *p* < 0.05, **Table 4**).

**Figure 4:**
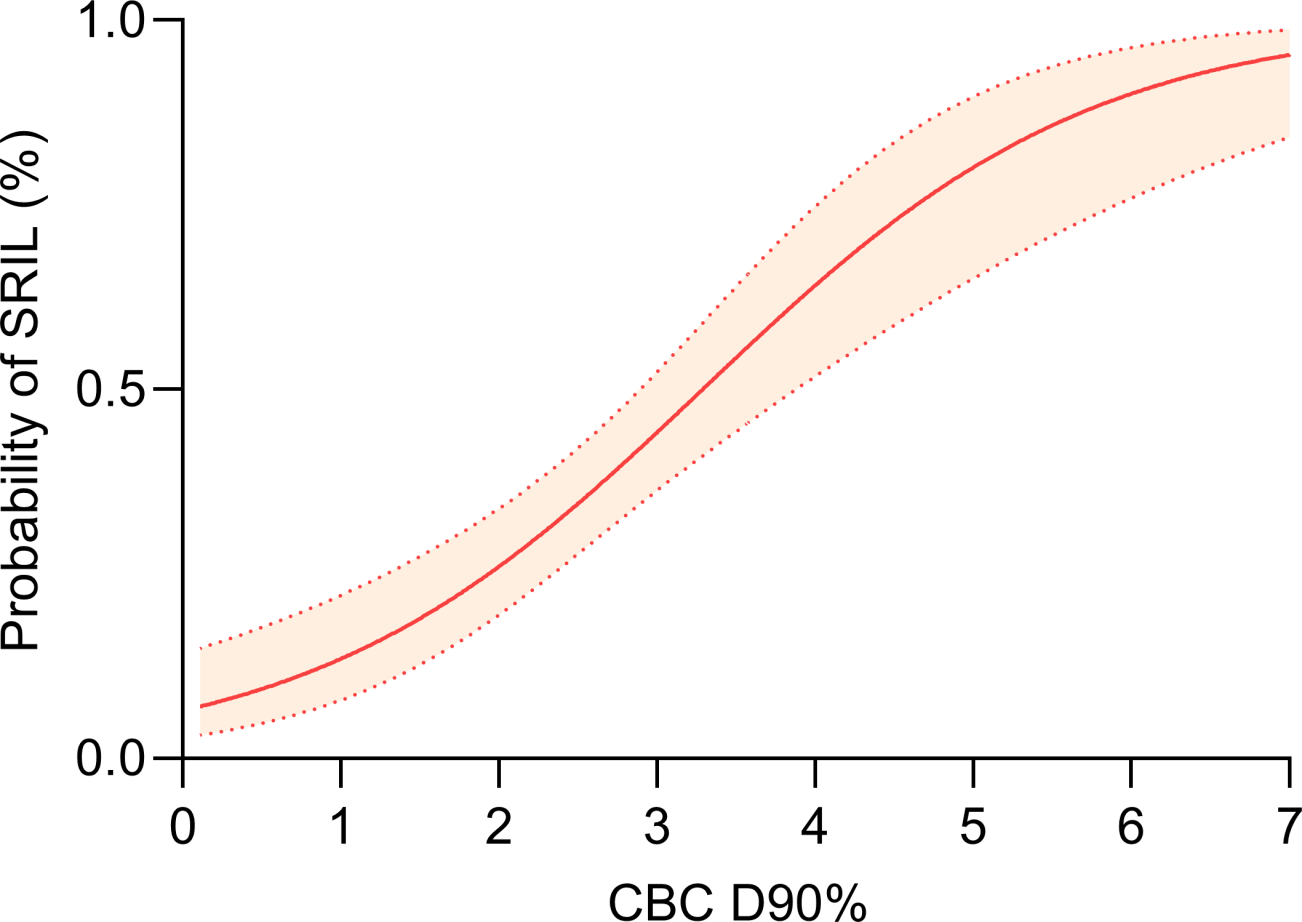
Dose-response relationship between dose to 90% of circulating blood cells and severe radiation-induced lymphopenia.

A dose-volume relationship between CBC D90% and the probability of SRIL was observed (**Figure 4**). In addition, the number of grade 4 lymphopenia events and CBC D90% were positively correlated (**Supplementary Figure 3**). When analyzing factors affecting D90% > 2.6 GyE, PBSPT significantly satisfied the dose criteria for CBC D90% of 2.6 GyE (OR = 0.11, 95% CI: 0.03–0.31, *p* = 0.004, **Table 5**). Furthermore, CBC D90% > 2.6 GyE was associated with poorer OS and PFS outcomes after excluding SRIL from the multivariable analysis (**Supplementary Table 6**).

**Table 5:** Factors related to the dose to 90% of circulating blood cells.

Variables		Univariate analysis			Multivariate analysis		
		OR	95% CI	*p*-value	OR	95% CI	*p*-value
Sex	(Female vs. male)	1.00	0.51–2.00	0.996			
Age	(<65 vs. ≥ 65 years)	0.90	0.51–1.57	0.712			
Histology	(non-ADC vs. ADC)	0.67	0.38–1.18	0.165			
Clinical T-stage	(cT1–2 vs. cT3–4)	1.20	0.68–2.13	0.533			
Clinical N-stage	(cN2 vs. cN3)	0.97	0.54–1.75	0.927			
Baseline ALC	(Continuous)	1.15	0.80–1.67	0.443			
PTV (per 10 cc)	(Continuous)	1.03	1.02–1.04	<.001	1.03	1.02–1.05	<.001
Total prescribed dose	(>66 vs. ≤66 GyE)	1.15	0.45–3.10	0.771			
Treatment modality	(IMRT vs. PBSPT)	0.22	0.09–0.51	0.001	0.11	0.03–0.31	0.004

*The foreparts of parentheses were set as the reference group.

ADC, adenocarcinoma; ALC, absolute lymphocyte count; PTV, planning target volume; GyE, gray equivalent; IMRT, intensity-modulated radiotherapy; PBSPT, pencil-beam scanning proton therapy.

## 4 Discussion

In this study, we investigated the prognostic value of SRIL and the correlation of bDVH with the development of SRIL in patients with locally advanced NSCLC treated with CCRT. SRIL was associated with poorer OS and PFS outcomes compared with the control. Moreover, at CBC D90% > 2.6 GyE, the risk of SRIL significantly increased together with baseline ALC and PTV. Moreover, PBPST was a significant contributor to minimizing CBC D90%. In addition, CBC D90% had a prognostic value for OS and PFS outcomes. To the best of our knowledge, this was the first study to discover the clinical impact of CBCs in patients with NSCLC and the potential benefit of PBSPT in minimizing radiation exposure to CBCs compared with IMRT (photon).

The negative impact of treatment-related lymphopenia on treatment outcomes in NSCLC has been widely investigated in recent studies (12, 17–23). Upadhyay et al. systematically reviewed 14 studies involving patients with lung cancer and reported that severe lymphopenia increased the risk of death with a pooled hazard ratio of 1.59 (*p* < 0.001) and the risk of death/progression with a pooled hazard ratio of 2.1 (*p* < 0.001) (17). Considering the radiosensitivity of lymphocytes, thoracic RT, which inevitably irradiates highly vascularized lymphocyte-rich organs, such as the lungs and heart, is a major contributor to lymphopenia or SRIL (24). We had previously reported the prognostic value of SRIL in patients with NSCLC (12). Although we could not perform subgroup analyses for patients treated with immunotherapy owing to the small sample size, the clinical significance of SRIL in the context of immunotherapy for NSCLC was highlighted (18, 19, 21–23). Jing et al. discovered that SRIL (defined as ALC < 230/µL at the end of CCRT) disrupted survival benefits of maintenance therapy with durvalumab following CCRT (21). In addition to the development of SRIL, Cho et al. reported that recovery from SRIL at 3 months after CCRT was significantly related to PFS and OS outcomes in patients treated with maintenance immunotherapy (18). The dismal effect of SRIL might stem from the reduced systemic anti-tumor immune response by lymphocytes and depletion of tumor-infiltrating lymphocyte (7, 25). The depletion of CD4^+^ T cells from SRIL, which control cell-mediated immunity against tumors and exert anti-tumor effects on CD8^+^ T cells, could influence the prognosis (26).

In view of decreasing the incidence of SRIL, several studies suggested various dose-volume criteria for predicting SRIL (12, 17, 20, 23, 27–29). In addition to the baseline ALC and target volume, most studies revealed that the dose to the lung or heart was predictive of SRIL (17). In this context, we had previously reported that lung V5Gy (OR = 1.07) and baseline ALC (OR = 0.73) were independent predictive factors of SRIL in 223 patients with NSCLC treated with CCRT (12). However, a dose–volume correlation in the lung or heart only indirectly provides the potential impact of RT on SRIL. Joseph et al. reported a negative correlation between the integral body dose and post-RT ALC in patients with lung cancer (29). Furthermore, neither dose to the lungs nor heart was significantly related to post-RT ALC. In addition, several reports highlighted that the effective dose to circulating immune cells (EDIC), which incorporates the mean lung dose, mean heart dose, and integral dose, was related to SRIL in lung, breast, and esophageal cancers (29–31). However, the EDIC equation was formulated based on limited patient data, i.e., patients receiving IMRT with over 25 fractions, and built for thoracic radiation fields only. The EDIC equation is yet to be validated with proton patient data. We chose HEDOS over EDIC in this study because using HEDOS, we can calculate the dose to CBCs utilizing the organ DVH from any treatment modality or treatment site. With a patient-specific bDVH from HEDOS results, the various DVH metrics were tested to find the most significant dosimetric factors for SRIL that could be used to guide the treatment plan in a way to reduce the SRIL risk. The last feature we employed in HEDOS was the dose rates from beam-on-times during beam delivery. Therefore, dose criteria for CBCs could be considered when planning for patients scheduled to receive immunotherapy.

Proton beam therapy is advantageous over photon RT in reducing the low-dose radiation to out-of-field. Based on the aforementioned evidence of the dose-response relationship between the healthy organs and SRIL, proton beam therapy is considered to be a potential therapeutic tool for lymphocyte-sparing RT. PBPST alleviates the risk of SRIL by reducing the lung V5Gy compared with IMRT in patients with NSCLC (12). The clinical significance of proton beam therapy in reducing SRIL has also been explored in primary brain tumors and esophageal cancer (32–34). However, to the best of our knowledge, this is the first analysis determining the clinical significance of PBSPT when incorporated with the dose calculation of CBCs. PBPST could positively affect the dose distribution to CBCs compared with IMRT. Furthermore, FLASH RT with an ultra-high dose-rate could enhance the immune-related tumor response by minimizing radiation to CBCs and subsequently reducing SRIL.

This study has several limitations. First, this study was associated with inherent limitations owing to its retrospective design. Second, the limited number of patients in the PBSPT group might have led to an overestimation of the effect of PBSPT. However, the target volume or baseline ALC, which could affect the SRIL development, did not differ significantly. Other limitations related to HEDOS calculations are using a uniform blood path distribution for all patients, implying that we used same the blood flow rate and blood volume for all patients, as these values were unavailable and impossible to measure in this retrospective study. We did not consider the realistic time structure of the beam delivery of IMRT or PBSPT. As these beams consist of many beamlets with a high dose to a small irradiation area, the realistic beam may produce a few CBCs irradiated with very high dose rates, thereby changing the bDVH shapes. The impact of the realistic time structure on the dose to CBCs should be further investigated. Further analyses are required to develop a model for predicting healthy tissue complication probability for SRIL based on CBCs.

The occurrence of SRIL was associated with an increased risk of tumor progression and death in patients with NSCLC treated with CCRT. We proposed and clinically validated the significance of bDVH in predicting SRIL. CBC D90% > 2.6 GyE significantly increased the risk of SRIL, and PBPST could decrease CBC D90% compared to IMRT. This analysis should be further validated through randomized controlled trials comparing PBPST and IMRT, particularly providing evidence of lymphocyte-sparing from the sparing dose to CBCs in the setting of maintenance immunotherapy.

## Data availability statement

The raw data supporting the conclusions of this article will be made available by the authors, without undue reservation.

## Ethics statement

The studies involving human participants were reviewed and approved by Samsung medical center. Written informed consent for participation was not required for this study in accordance with the national legislation and the institutional requirements.

## Author contributions

Conception, design, data collection, interpretation, and drafting of the manuscript were performed by NK, JS, and SA. Statistical analysis and editing of the manuscript were performed by NK and JS. All authors contributed to the article and approved the submitted version.
