# Supplementary material for: Reduced radiation exposure to circulating blood cells in proton therapy compared with X-ray therapy in locally advanced lung cancer: Computational simulation based on circulating blood cells
Source: Front Oncol. 2023 Feb 27;13:1119173. doi: 10.3389/fonc.2023.1119173 (PMC10009224; doi:10.3389/fonc.2023.1119173)
Supplement: Supplementary file 1 [file DataSheet_1.docx]

Supplementary Material

Reduced radiation exposure to circulating blood cells in proton therapy compared with X-ray therapy in locally advanced lung cancer: computational simulation based on circulating blood cells

**Nalee Kim, MD, PhD^1+*^, Jungwook Shin, PhD^2+^, Sung Hwan Ahn PhD^1+^, Hongryull Pyo, MD, PhD^1^, Jae Myoung Noh, MD, PhD^1^, Yong Chan Ahn, MD, PhD^1^, Kyungmi Yang, MD, PhD^1^, Woojin Lee, PhD^1^ and Byoungsuk Park, MS^1^**

^1^Department of Radiation Oncology, Samsung Medical Center, Sungkyunkwan University School of Medicine, Republic of Korea

^2^Division of Cancer Epidemiology and Genetics, National Cancer Institute, National Institutes of Health, Rockville, MD 20850, United States of America

^+^N. Kim, J. Shin, and S. H. Ahn contributed equally to this work.

*** Correspondence:**Nalee Kim, MD, PhD

Department of Radiation Oncology, Samsung Medical Center, Sungkyunkwan University School of Medicine, 81 Irwon-ro, Gangnam-gu, Seoul 06351, Republic of Korea

Tel.: 82-2-3410-2612; Fax: 82-2-3410-2619; Email: rodr.naleekim@gmail.com

Keywords: Proton beam therapy, Lung cancer, Lymphopenia, Radiation therapy, Blood

**Supplementary Table 1** Dose constraints for organs-at-risk.

| Organ at risk | Dose constraints |
| --- | --- |
|  |  |
| Spinal cord | Dmax <45 GyE |
| Both lungs | Dmean <20 GyE |
|  | V20GyE<35% |
|  | V10GyE<45% |
|  | V10GyE<65% |
| Heart | V40GyE < 50% |
| Esophagus | Dmax < 66 GyE |
|  | Dmean < 33 GyE |
|  | V45GyE < 50% |

*Abbreviations*: GyE, gray equivalent; Dmax, maximum dose; Dmean, mean dose; VXXGyE, volume receiving more than XX GyE.

**Supplementary Table 2** Baseline characteristics according to the treatment modality.

|  |  | **IMRT** | **PBSPT** | ***p*-value** |
| --- | --- | --- | --- | --- |
|  |  | **n = 164** | **n = 37** |  |
| Sex | Female | 37 (22.6) | 5 (13.5) | 0.221 |
|  | Male | 127 (77.4) | 32 (86.5) |  |
| Age |  | 63 [56–68] | 67 [62–74] | 0.001 |
| ECOG | 0 | 21 (12.8) | 7 (18.9) | 0.332 |
|  | 1 or 2 | 143 (87.2) | 30 (81.1) |  |
| Smoking history | Never-smoker | 36 (22.0) | 3 (8.1) | 0.054 |
|  | Ex- or current-smoker | 128 (78.0) | 34 (91.9) |  |
| Tumor laterality | Left | 63 (38.4) | 15 (40.5) | 0.841 |
|  | Right | 95 (57.9) | 20 (54.1) |  |
|  | Central | 6 (3.7) | 2 (5.4) |  |
| Tumor location | Upper lobe | 92 (56.1) | 19 (51.4) | 0.284 |
|  | Middle lobe | 16 (9.8) | 7 (18.9) |  |
|  | Lower lobe | 56 (34.1) | 11 (29.7) |  |
| Pathology | Non-ADC | 69 (42.1) | 24 (64.9) | 0.012 |
|  | ADC | 95 (57.9) | 13 (35.1) |  |
| T-stage | cT1 or cT2 | 102 (62.2) | 22 (59.5) | 0.757 |
|  | cT3 or cT4 | 62 (37.8) | 15 (40.5) |  |
| N-stage | cN2 | 49 (29.9) | 21 (56.8) | 0.002 |
|  | cN3 | 115 (70.1) | 16 (43.2) |  |
| Clinical stage | IIIA | 18 (11.0) | 14 (37.8) | <0.001 |
|  | IIIB | 118 (72.0) | 12 (32.4) |  |
|  | IIIC | 28 (17.1) | 11 (29.7) |  |
| Baseline ALC | (×10^3^/μL) | 2.05 [1.62–2.54] | 2.13 [1.61–2.34] | 0.844 |

Values are expressed as number of patients (%) or median [interquartile range].

Abbreviations: IMRT, intensity-modulated radiotherapy; PBSPT, pencil-beam scanning proton therapy; ECOG, Eastern Cooperative Oncology Group; ADC, adenocarcinoma.

**Supplementary Table 3** Absolute lymphocyte count during concurrent chemoradiation therapy according to the treatment modality.

|  |  | **Total** | **IMRT** | **PBSPT** | ***p*-value** |
| --- | --- | --- | --- | --- | --- |
| **ALC (×10^3^/uL)** |  | **n = 201** | **n = 164** | **n = 37** |  |
| Baseline | ALC | 2.07 [1.62–2.51] | 2.05 [1.62–2.54] | 2.13 [1.61–2.34] | 0.844 |
| During CCRT | 1^st^ week of CCRT | 1.14 [0.78–1.53] | 1.15 [0.76–1.54] | 1.14 [0.94–1.52] | 0.572 |
|  | 2^nd^ week of CCRT | 0.63 [0.45–0.86] | 0.61 [0.42–0.81] | 0.79 [0.59–0.98] | 0.003 |
|  | 3^rd^ week of CCRT | 0.40 [0.29–0.59] | 0.40 [0.27–0.58] | 0.42 [0.34–0.62] | 0.211 |
|  | 4^th^ week of CCRT | 0.33 [0.21–0.50] | 0.31 [0.20–0.46] | 0.43 [0.27–0.60] | 0.014 |
|  | 5^th^ week of CCRT | 0.28 [0.16–0.43] | 0.26 [0.15–0.40] | 0.41 [0.28–0.55] | <.001 |
|  | Last week of CCRT | 0.28 [0.16–0.46] | 0.25 [0.15–0.45] | 0.38 [0.26–0.50] | 0.005 |
| Post-CCRT | Post-CCRT 1 month | 1.02 [0.65–1.53] | 1.00 [0.64–1.58] | 1.08 [0.77–1.48] | 0.775 |
|  | Post-CCRT 3 months | 1.19 [0.88–1.56] | 1.21 [0.89–1.62] | 1.14 [0.82–1.41] | 0.230 |
|  | Post-CCRT 6 months | 1.31 [0.92–1.71] | 1.29 [0.98–1.65] | 1.44 [0.85–1.88] | 0.504 |
|  |  |  |  |  |  |
| During CCRT | Grade 4 Lymphopenia | 107 (53.2) | 94 (57.3) | 13 (35.1) | 0.015 |
| Number of events | 0 | 94 (46.8) | 70 (42.7) | 24 (64.9) | 0.024 |
|  | 1 | 35 (17.4) | 26 (15.9) | 9 (24.3) |  |
|  | 2 | 52 (25.9) | 48 (29.3) | 4 (10.8) |  |
|  | 3 | 16 (8.0) | 16 (9.8) | 0 (0.0) |  |
|  | 4 | 2 (1.0) | 2 (1.2) | 0 (0.0) |  |
|  | 5 | 2 (1.0) | 2 (1.2) | 0 (0.0) |  |

*Abbreviations*: IMRT, intensity-modulated radiotherapy; PBSPT, pencil-beam scanning proton therapy; CCRT, concurrent chemoradiation therapy.

**Supplementary Table 4** Univariate analysis to predict severe radiation-induced lymphopenia including dose parameters.

| **Variables** |  | **Univariate analysis** | | |
| --- | --- | --- | --- | --- |
|  |  | **OR** | **95% CI** | ***p*-value** |
| Sex | (Female vs. male) | 0.78 | 0.39–1.58 | 0.480 |
| Age | (<65 vs. ≥65 years) | 1.01 | 0.56–1.80 | 0.972 |
| Histology | (non-ADC vs. ADC) | 1.12 | 0.63–2.01 | 0.698 |
| Clinical T-stage | (cT1–2 vs. cT3–4) | 0.95 | 0.52–1.71 | 0.860 |
| Clinical N-stage | (cN2 vs. cN3) | 1.65 | 0.89–3.12 | 0.119 |
| Contralateral mediastinal node | (No vs. yes) | 1.14 | 0.63–2.05 | 0.668 |
| Supraclavicular node | (No vs. yes) | 1.51 | 0.82–2.74 | 0.186 |
| Baseline ALC | (Continuous) | 0.48 | 0.30–0.74 | 0.002 |
| PTV | (Continuous, per 10 cc) | 1.02 | 1.01–1.03 | <.001 |
| PTV | (<500 vs. ≥500) | 4.00 | 2.14–7.78 | <.001 |
| Total prescribed dose | (>66 vs. ≤66 GyE) | 1.23 | 0.46–3.65 | 0.686 |
| CBC mean dose, GyE | (Continuous) | 1.93 | 1.53–2.53 | <.001 |
| CBC D10, GyE | (Continuous) | 1.75 | 1.43–2.21 | <.001 |
| CBC D20, GyE | (Continuous) | 1.81 | 1.46–2.30 | <.001 |
| CBC D30, GyE | (Continuous) | 1.85 | 1.48–2.38 | <.001 |
| CBC D40, GyE | (Continuous) | 1.89 | 1.50–2.45 | <.001 |
| CBC D50, GyE | (Continuous) | 1.94 | 1.53–2.53 | <.001 |
| CBC D60, GyE | (Continuous) | 1.98 | 1.55–2.61 | <.001 |
| CBC D70, GyE | (Continuous) | 2.04 | 1.58–2.71 | <.001 |
| CBC D80, GyE | (Continuous) | 2.11 | 1.61–2.84 | <.001 |
| CBC D90, GyE | (Continuous) | 2.21 | 1.67–3.04 | <.001 |
| CBC V_0.5GyE_, % | (Continuous) | 1.32 | 1.00–6.94 | 0.508 |
| CBC V_1.0GyE_, % | (Continuous) | 1.06 | 1.01–1.16 | 0.109 |
| CBC V_1.5GyE_, % | (Continuous) | 1.02 | 1.01–1.04 | 0.005 |
| CBC V_2.0GyE_, % | (Continuous) | 1.02 | 1.01–1.03 | 0.003 |
| CBC V_2.5GyE_, % | (Continuous) | 1.02 | 1.01–1.03 | <.001 |
| CBC V_3.0GyE_, % | (Continuous) | 1.02 | 1.01–1.03 | <.001 |
| CBC V_3.5GyE_, % | (Continuous) | 1.02 | 1.01–1.03 | <.001 |

*The foreparts of parentheses were set as the reference group.

*Abbreviations*: ADC, adenocarcinoma; ALC, absolute lymphocyte count; PTV, planning target volume; GyE, gray equivalent; CBC, circulating blood cell; Dxx, dose to XX% of volume; VXXGyE, volume receiving over XX GyE.

**Supplementary Table 5** Construction of the final multivariate model to predict the severe radiation-induced lymphopenia risk based on the dose to circulating blood cells.

| Model variables | AIC |
| --- | --- |
| Baseline ALC + PTV (Null model) | 245.52 |
| Null model + Dmean > 3.1 GyE | 227.32 |
| Null model + D10 > 3.9 GyE | 237.14 |
| Null model + D30 > 3.3 GyE | 228.52 |
| Null model + D50 > 3.1 GyE | 227.32 |
| Null model + D70 > 2.8 GyE | 232.92 |
| Null model + D90 > 2.6 GyE | 218.46 |
| Null model + V_1.5GyE_ > 99% | 244.63 |
| Null model + V_2.0GyE_ > 99% | 233.40 |
| Null model + V_2.5GyE_ > 95% | 226.41 |
| Null model + V_3.0GyE_ > 71% | 228.26 |
| Null model + V_3.5GyE_ > 20% | 228.36 |

*Abbreviations*: AIC, Akaike information criterion; ALC, absolute lymphocyte count; PTV, planning target volume; GyE, gray equivalent; Dxx, dose to XX% of volume; V_XXGyE_, volume receiving more than XX GyE.

**Supplementary Table 6.** Prognostic factors for overall and progression-free survival.

| **Overall survival** |  | **Univariable analysis** | | | **Multivariable analysis** | | |
| --- | --- | --- | --- | --- | --- | --- | --- |
| **Variables** |  | **HR** | **95% CI** | ***p*-value** | **HR** | **95% CI** | ***p*-value** |
| Treatment modality | (IMRT vs. PBSPT) | 0.84 | 0.46–1.50 | 0.551 |  |  |  |
| Sex | (Female vs. male) | 1.62 | 0.94–2.78 | 0.083 |  |  |  |
| Age | (<65 vs. ≥65 years) | 1.18 | 0.78–1.77 | 0.442 |  |  |  |
| Histology | (non-ADC vs. ADC) | 0.52 | 0.35–0.79 | 0.002 | 0.59 | 0.39–0.90 | 0.014 |
| Clinical T-stage | (T1–2 vs. T3–4) | 1.18 | 0.78–1.79 | 0.444 |  |  |  |
| Clinical N-stage | (N2 vs. N3) | 0.73 | 0.48–1.12 | 0.151 |  |  |  |
| GTV | Continuous (per 10cc) | 1.33 | 1.12–1.57 | 0.001 | 1.01 | 0.99–1.02 | 0.187 |
| Total dose | (>66 vs. ≤66 GyE) | 1.56 | 0.72–3.37 | 0.263 |  |  |  |
| BED10 | (<80 vs. ≥80 GyE) | 1.20 | 0.73–1.95 | 0.469 |  |  |  |
| Baseline ALC | (continuous) | 0.41 | 0.20–1.11 | 0.137 |  |  |  |
| CBC D90 | (≤2.6 vs. >2.6 GyE) | 2.14 | 1.41–3.24 | <.001 | 1.92 | 1.25–2.94 | 0.003 |
| **Progression-free survival** |  | **Univariable analysis** | | | **Multivariable analysis** | | |
| **Variables** |  | **HR** | **95% CI** | ***p*-value** | **HR** | **95% CI** | ***p*-value** |
| Treatment modality | (IMRT vs. PBSPT) | 0.57 | 0.36–0.91 | 0.019 | 0.72 | 0.44–1.16 | 0.181 |
| Sex | (Female vs. male) | 0.84 | 0.57–1.21 | 0.344 |  |  |  |
| Age | (<65 vs. ≥ 65 years) | 0.63 | 0.45–0.87 | 0.005 | 0.62 | 0.45–0.87 | 0.006 |
| Histology | (non-ADC vs. ADC) | 1.08 | 0.78–1.48 | 0.651 |  |  |  |
| Clinical T-stage | (T1–2 vs. T3–4) | 0.98 | 0.71–1.35 | 0.881 |  |  |  |
| Clinical N-stage | (N2 vs. N3) | 1.11 | 0.79–1.55 | 0.550 |  |  |  |
| GTV | Continuous (per 10cc) | 1.01 | 1.00–1.02 | 0.142 |  |  |  |
| Total dose | (>66 vs. ≤66 GyE) | 1.09 | 0.63–1.88 | 0.768 |  |  |  |
| BED10 | (<80 vs. ≥80 GyE) | 1.43 | 0.98–2.10 | 0.065 |  |  |  |
| Baseline ALC | (continuous) | 0.75 | 0.60–0.93 | 0.009 | 1.47 | 1.02–2.13 | 0.040 |
| CBC D90 | (≤2.6 vs. >2.6 GyE) | 1.72 | 1.25–2.37 | 0.001 | 1.68 | 1.21–2.35 | 0.002 |

*The foreparts of parentheses were set as the reference group.

*Abbreviations*: HR, hazard ratio; CI, confidence interval; IMRT, intensity-modulated radiotherapy; PBSPT, pencil-beam scanning proton therapy; ADC, adenocarcinoma; GTV, gross tumor volume; GyE, gray equivalent; BED10, biological effective dose with α/β of 10; ALC, absolute lymphocyte count; CBC D90, dose to 90% of circulating blood cells.

**Supplementary Figure 1**

CONSORT flow diagram.


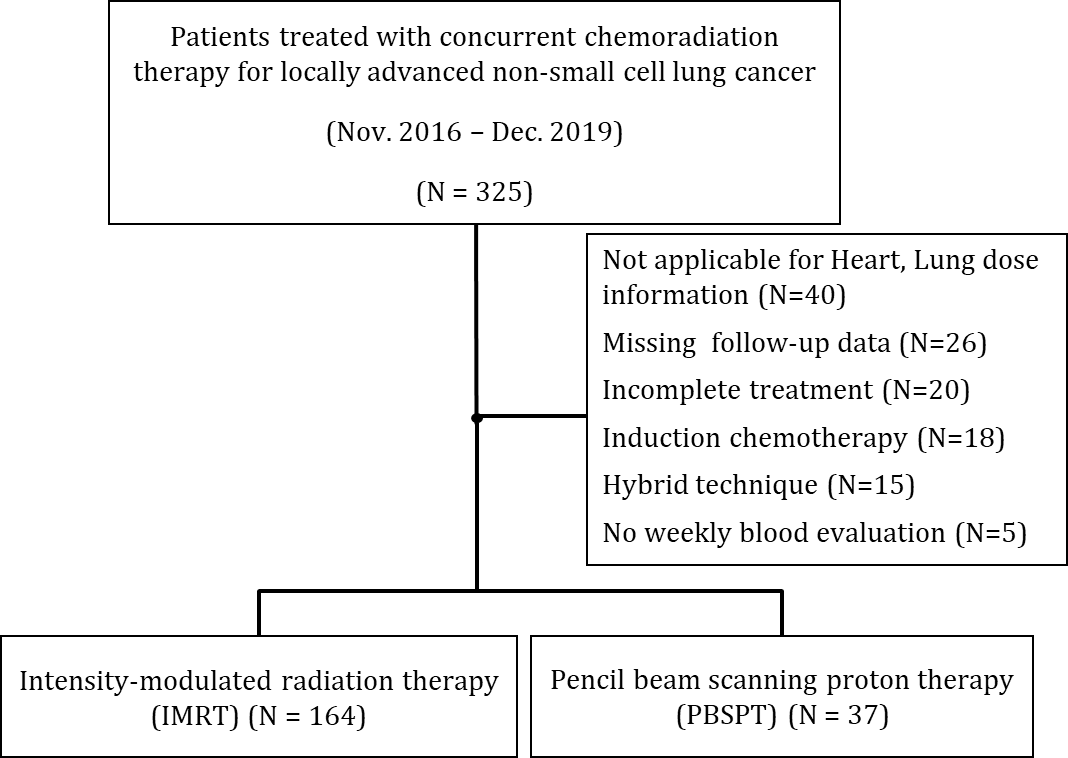


**Supplementary Figure 2**

Decrease in the absolute lymphocyte count during and after concurrent chemoradiation therapy according to the treatment modality.


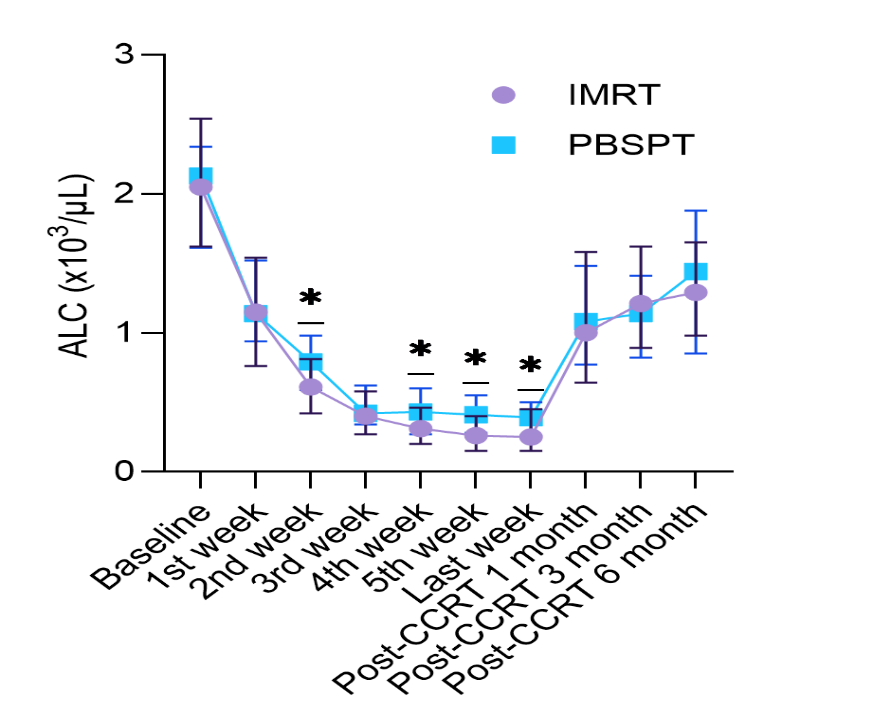


Abbreviations: ALC, absolute lymphocyte count; CCRT, concurrent chemoradiation therapy; IMRT, intensity-modulated radiotherapy; PBSPT, pencil-beam scanning proton beam therapy.

*Statistically significant, *p* < 0.05.

**Supplementary Figure 3**

Dose to 90% of circulating blood cell according to the number of grade 4 lymphopenia events.
